# Analysis of the Effect of Antenna-to-Head Distance for Microwave Brain Imaging Applications

**DOI:** 10.1155/ijbi/8872566

**Published:** 2025-05-04

**Authors:** Farhana Parveen, Parveen Wahid

**Affiliations:** ^1^Department of Electrical and Electronic Engineering, East West University, Dhaka, Bangladesh; ^2^Department of Electrical and Computer Engineering, University of Central Florida, Orlando, Florida, USA

**Keywords:** microwave brain imaging, reflection coefficient, specific absorption rate (SAR), surface current distribution, Vivaldi antenna

## Abstract

Wideband antennas are extensively used in many medical applications, which require the placement of the antenna on or near a human body. The performance of the antenna should remain compliant with the requirements of the target application when placed in front of the subject under investigation. Since the performance of an antenna varies when the distance from the subject is changed, the effect of varying the distance of a miniaturized wideband antipodal Vivaldi antenna from a numerical head model is analyzed in this work. The analyses can demonstrate whether the antenna performance and its effect on the head aptly comply with the requirements for the intended application of microwave brain imaging. It is observed that, when the antenna-head distance is increased, the background noise in the received signal is enhanced, whereas when the distance is reduced, the radiation-safety consideration on the head is affected. Hence, the optimum distance should provide a good compromise in terms of both signal receptibility by the antenna and radiation safety on the head. As the optimum antenna-to-head distance may vary with the change in antenna, measurement system, and the surrounding medium, this work presents a basic analysis procedure to find the appropriate antenna distance for the intended application.

## 1. Introduction

Many medical applications, such as microwave imaging, hyperthermia, and remote monitoring/implantable devices [1–3], require wideband antennas as a transmitter and/or a receiver of electromagnetic waves. The design and performance analysis of these antennas are carried out based on application-specific standards and requirements [4–7]. These include complying with the desired performance not only in a standalone setup but also in the target environment of the respective application. The performance of an antenna is greatly impacted when it is placed close to an object/subject under investigation, and it will differ from the case when it is working alone in the air medium.

In this context, the effect of a miniaturized wideband antipodal Vivaldi antenna [8], while placed in front of a numerical head model, is investigated in this work for the application of microwave imaging of the brain. The performance criteria used for the investigation are selected based on signal receptibility, noise reduction, and safety considerations. As reported in [8], the design procedure of the antenna under investigation involved analysis and testing of the antenna in a standalone setup while placed in the air medium. In [9, 10], the application of the antenna was demonstrated in the case of microwave imaging of the head for the detection of a blood clot inside the brain, where the antenna was placed in front of a head model in an air medium (i.e., no matching liquid was used). However, no analyses were reported in these works [9, 10] on the effect of varying the antenna-to-head distance on the antenna or the head. Hence, the work in this paper is aimed at presenting an analysis of the effects of the distance between the antenna and the head model on both the antenna performance (in terms of surface current distribution, input impedance, and reflection coefficient, i.e., received signal and antenna reverberation) and the head model (in terms of specific absorption rate (SAR), field penetration depth, etc.).

When the head model is moved further away from the antenna, the reflected signal needs to travel a longer distance to reach back to the antenna. This increases noise exposure in the received signal. Furthermore, due to wave spreading, a fraction of the reflected signal gets lost in the medium between the antenna and the head. On the other hand, when the head model is moved closer to the antenna, the artifacts due to skull reflections and antenna reverberations become more pronounced in the received signal. The large skull reflections and antenna reverberations overlap the reflections from shallow anomalies, making the image reconstruction process more difficult. Furthermore, placing the head closer to the antenna increases the SAR in the brain, thus making the radiation-safety considerations more prominent. These effects may vary with the change in antenna parameters, type of object, operating frequency range, measurement setup, and/or the surrounding medium. Hence, the investigation of the distance between the antenna and the head is essential for obtaining the most optimized signal receptibility by reducing the exposure to background noise, skull reflections, antenna reverberations, and other artifacts while ensuring radiation safety on the head model.

In [9, 10], the antenna is placed at a distance of 4.5 cm from the head model. However, several works in literature [11–15] have demonstrated the feasibility of near-field microwave imaging using different antennas and imaging algorithms, where the subject under investigation (e.g., breast, brain, bone, and lungs) is kept in close proximity to the antenna. In view of the promising aspects of near-field imaging demonstrated in these works, the effect of reducing the antenna-head distance, from that reported in [9, 10], is analyzed in this work on both the antenna and the head model. For the case of placing the head model closer to the antenna, the radiation safety considerations are of pivotal importance. Hence, the SAR in the head model is calculated to determine the maximum permissible stimulation power of the antenna when it is placed in close proximity to the head. A safe operation can be ensured when the object is exposed to nonionizing radiation as specified in the ICNIRP [16] and IEEE (*IEEE Std C95.1*) standards [17]. On the other hand, due to the wide beamwidth of the antenna under investigation [8], the wave spreading loss deteriorates as the distance between the antenna and the head is increased. This causes a significant loss of target information from the head interior. Furthermore, the reflected signal gets exposed to more background noise due to the increased distance while travelling back toward the antenna. Hence, in this work, the antenna-head distance is not increased beyond that reported in [9, 10] to ensure lower wave spreading loss.

The paper is organized as follows: Section 2 introduces the antenna under investigation and verifies CST simulation results with respect to HFSS simulation results and measurement results.  Section 3 presents the analysis of varying the antenna-to-head distance and the investigations of the effects on antenna performance and head model in terms of signal receptibility and radiation safety, respectively.  Section 4 presents the analysis of the antenna parameters for optimizing the antenna for the intended application by reducing the antenna reverberations for a particular antenna-to-head distance. Finally, Section 5 contains the conclusions of the analyses.

## 2. The Antenna Under Investigation: A Miniaturized Antipodal Vivaldi Antenna

**Figure 1:**
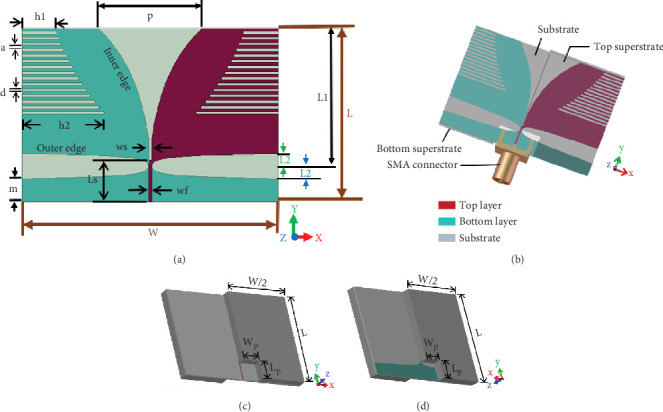
Antenna under investigation: (a) without superstrates [8], (b) with superstrates, and (c, d) position and dimensions of the top and bottom superstrates, respectively (*L* = 30.2 mm, *W* = 44.4 mm, *a* = *d* = 0.5 mm, *h*1 = 6 mm, *h*2 = 14.27 mm, *L*1 = 24 mm, *L*2 = 2.2 mm, *p* = 18 mm, *wf* = 0.5 mm, *m* = 4 mm, *ws* = 0.5 mm, *Ls* = 7.2 mm, *Wp* = 6 mm, and *Lp* = 6 mm).

Figure 1 shows the top view and isometric view, along with the design parameters, of the antenna under investigation. The antenna is designed using Rogers RT6010 (relative dielectric constant, *ɛ*_*r*_ = 10.2; thickness, *h* = 0.64 mm) as the substrate. The key design features of this antenna are the use of an elliptical tapered slot, feed slot, corrugations, and superstrates. The total length (*L*) and width (*W*) of the antenna are 30.2 and 44.4 mm, respectively. The antenna is fed with a 50 Ω microstrip line. The antenna investigated in this work is obtained from the antenna proposed in [8] by cutting a slot from each superstrate, near the feed region, to accommodate the SMA connector.  Figure 1a shows the antenna without the superstrates.  Figure 1b shows the antenna with the modified superstrates along with the SMA connector. The position and dimensions of the top and bottom superstrates are shown in Figure 1c,d, respectively.

The antenna model is simulated using both HFSS and CST Microwave Studio time-domain (TD) and frequency-domain (FD) solvers. The results reported in [8] were obtained from simulation using HFSS, whereas the analyses reported in this paper for the investigation of the antenna-to-head distance on the application of microwave imaging are obtained from CST simulations. Hence, in this section, the accuracy of the CST simulation results is verified by comparing the CST results with HFSS simulation and measurement results [8] to justify the validity of the analyses presented in the next section, which are based on the results obtained from the CST TD simulation.

**Figure 2:**
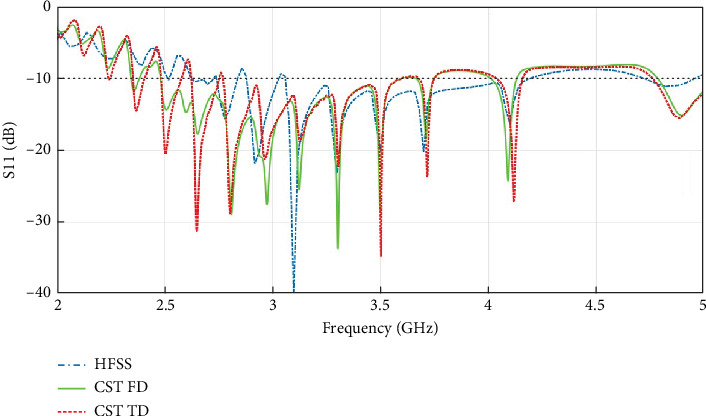
Reflection coefficient (*S*_11_) of the antenna obtained from HFSS, CST time-domain, and CST frequency-domain simulations.

Figure 2 shows the reflection coefficient (*S*_11_) plots of the antenna, obtained from HFSS, CST TD, and CST FD solvers. In this work, adaptive mesh refinement is applied in each solver to ensure accuracy. For CST, hexahedral and tetrahedral meshing are used in the model for TD and FD simulations, respectively. An open (PML) boundary is assigned at a distance greater than half a wavelength at the lowest simulation frequency (2 GHz) from each side of the antenna. The antenna is stimulated through the SMA connector using a waveguide port. The S-parameter (*S*_11_) plot shows reasonable agreement among different simulators. In general, from the simulation results, *S*_11_ < −10 dB is obtained within the frequency range of around ~2.65–4.2 GHz.

**Figure 3:**
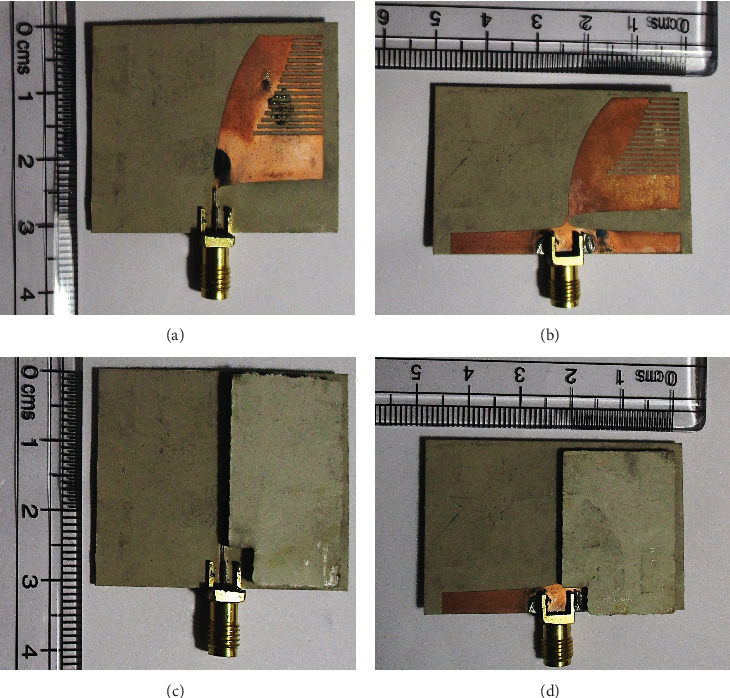
Fabricated prototype of the antenna: (a) top view (without superstrate), (b) bottom view (without superstrate), (c) top view (with superstrate), and (d) bottom view (with superstrate) [8].

**Figure 4:**
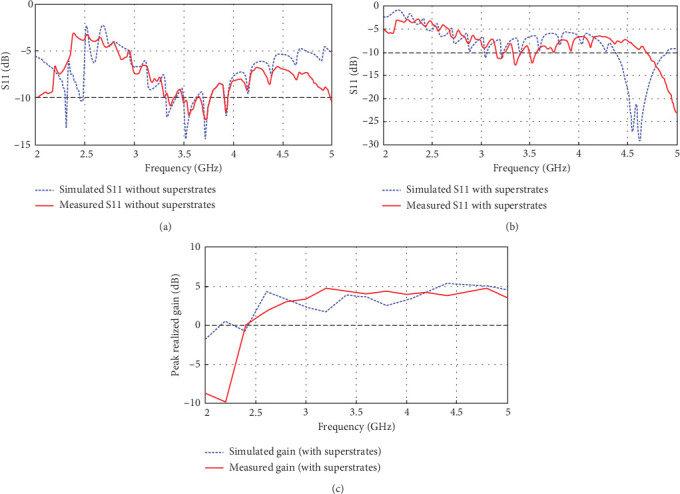
Comparison of CST time-domain simulation results with measurement results: (a, b) reflection coefficient (*S*_11_) without and with superstrates and (c) peak realized gain.

Figure 3 shows the top and bottom views of the fabricated prototype of the antenna with and without the superstrates. The reflection coefficient (*S*_11_) and gain measurements are done inside an anechoic chamber using an N5230A PNA-L [8]. The CST simulation results are compared with the measurement results in Figure 4. The CST antenna model is modified to mimic the defects incurred during the fabrication process, such as a slight misalignment between the top and bottom copper layers and the insertion of a thin air gap between the substrate and superstrates [4]. It can be seen from Figure 4 that the CST TD simulation results are in close agreement with the measured *S*_11_ and gain. This implies the extent of accuracy and reliability of the CST simulation results.

## 3. Analysis of the Effect of Varying the Distance Between the Antenna and the Head Model

The design of the antenna under investigation [8] was done based on the performance in a standalone setup in the air medium. However, when placed in close proximity to a head model, the performance of the antenna will be altered. In this section, the antenna performance is analyzed in terms of the surface current distribution and input impedance while the antenna is placed in front of a numerical head model.

**Figure 5:**
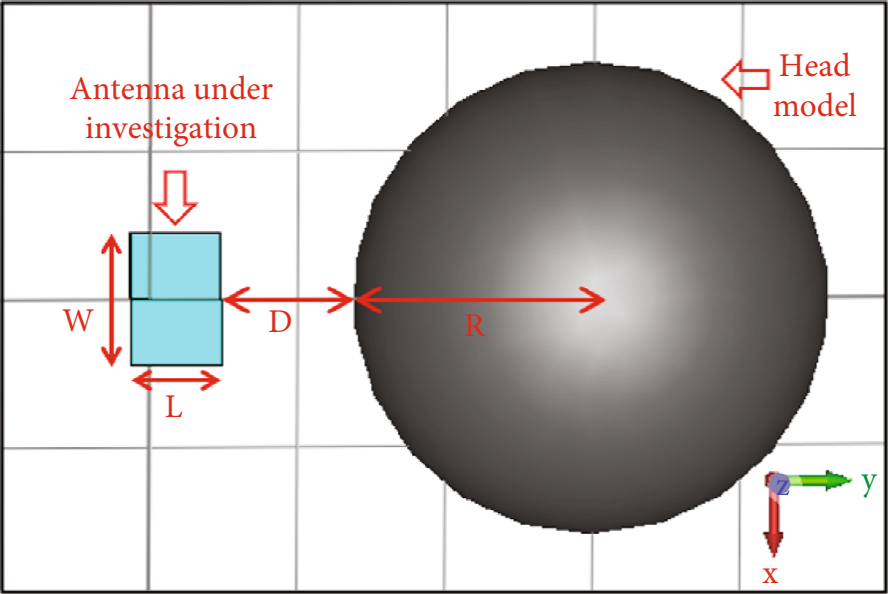
Antenna position in front of the numerical head model. *D* = Distance of the head model from the antenna (*W* = 44.4 mm, *L* = 30.2 mm, and *R* = 80 mm).

Figure 5 shows the placement of the antenna in air (no matching liquid) in front of the head at a distance *D*. The head is numerically modeled as a sphere of radius 8 cm with a homogeneous region having a dielectric constant of *ɛ*_*r*_ = 40, dielectric loss tangent of tan*δ* = 0.27653, and mass density = 1042.67 kg/m^3^ [4, 18, 19]. These parameters are held constant throughout the range of operating frequencies (2.8–4 GHz). The analysis of the effect of the distance between the antenna and the head model is made by placing the head model at three different distances from the antenna, having *D* = 1, 2, and 4.5 cm, which correspond to approximately *λ*/10, *λ*/5, and *λ*/2, respectively, where *λ* is the wavelength at the lowest frequency of operation (2.8 GHz).

### 3.1. Analysis of the Effects on the Antenna

The effects of varying the distance between the antenna and the head are analyzed in terms of antenna surface current distribution and input impedance at the three distances mentioned.

**Figure 6:**
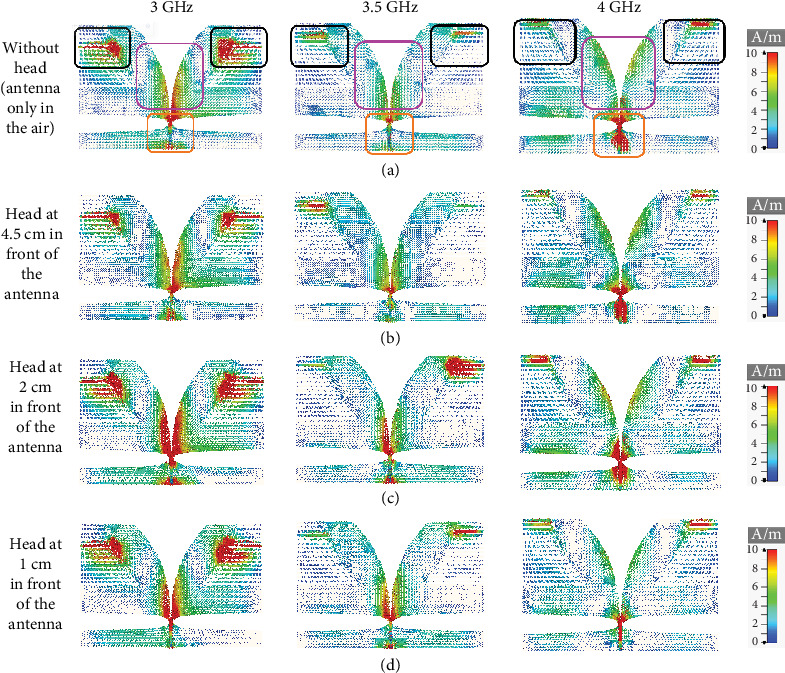
(a–d) Surface current distribution of the antenna at different frequencies within the operating frequency band for various antenna-to-head distances.

Figure 6 shows the effect of the distance (*D*) of the head model on the antenna surface current distribution at different frequencies within the operating frequency range. The current distribution on the antenna surface is shown in Figure 6a with no head model present (i.e., the antenna is placed alone in the air medium) and in Figures 6b, 6c, and 6d with the head model present at a distance of *D* = 4.5, 2, and 1 cm, respectively. It can be seen from Figure 6 that the effect of the head model on the surface current distribution is more pronounced when it is placed at the distance of *D* = 1 and 2 cm than at the distance of *D* = 4.5 cm. For example, the observation of the feed region (encircled by an orange border in Figure 6a) reveals that when the head model is placed at a distance of 4.5 cm from the antenna, the surface current distribution is not much altered from the case when no head model is present in front of the antenna. However, the current distribution changes significantly when the head model is placed at a 1- or 2-cm distance from the antenna. This distinction in surface current distribution is also observable near the corrugation region (encircled by black borders in Figure 6a) and tapered slot edge region (encircled by a purple border in Figure 6a). This emphasizes the fact that the head model, when brought closer to the antenna, has a greater impact on the reflected signal received by the antenna.

**Figure 7:**
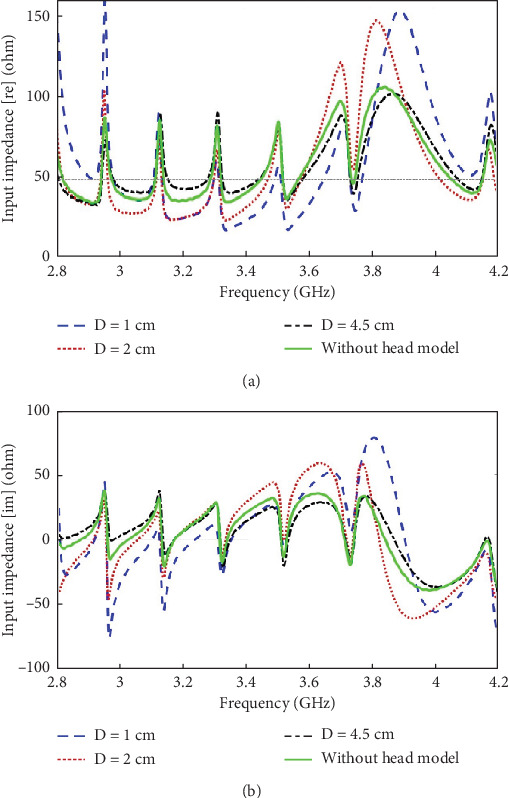
(a) Real part and (b) imaginary part of the input impedance of the antenna for various distances of the head model from the antenna as compared to the input impedance in a standalone setup of the antenna (i.e., when no head model is present).

The above observation is further supported by the input impedance plot of the antenna, as shown in Figure 7, for various distances of the head model from the antenna. When the head model is placed at a distance of *D* = 4.5 cm from the antenna, the input impedance is quite close to that when no head model is present. The closer the head model is brought to the antenna, the more changes are observed in the input impedance plot of the antenna. Hence, it is evident that the head model has a greater impact on the signal received at the antenna input port when it is placed at a distance of *D* = 1 and 2 cm than *D* = 4.5 cm.

### 3.2. Analysis of the Effects on the Head

The effects of varying the distance between the antenna and the head are analyzed in terms of field penetration profile and SAR distribution in the head.

**Figure 8:**
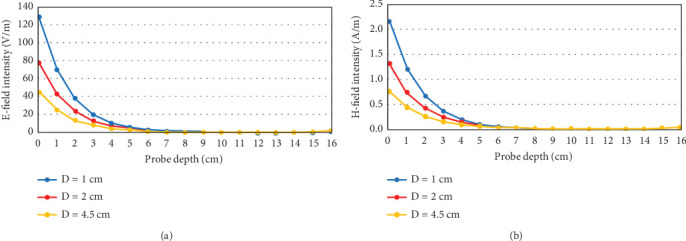
(a) E- and (b) H-field intensities at various depths inside the head model while the antenna is placed at different distances (*D*) from the head model.

The E- and H-field intensities with respect to time are plotted at several depths inside the head using E- and H-field probes in CST with 0.5 W antenna stimulation power.  Figure 8 shows the maximum (over time) E- and H-field intensities at various depths inside the head model. It can be seen that the field intensity is the highest at the head surface closest to the antenna (0 cm probe depth), and this intensity decreases as the antenna is moved further away from the head. Again, the field decays very sharply as it penetrates deeper into the head. More importantly, it is observed that the rate of decay in the field intensities is larger for smaller values of *D*; that is, the closer the antenna is placed to the head model, the faster the field decays as it travels inside the head.

**Figure 9:**
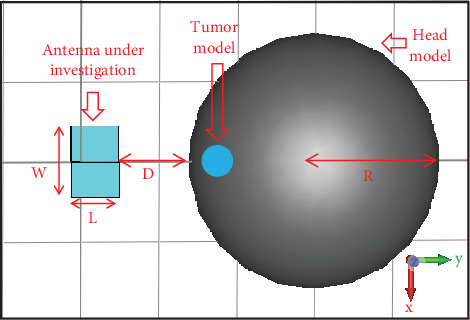
Tumor position inside the numerical head model (*D* = distance of the head model from the antenna, *W* = 44.4 mm, *L* = 30.2 mm, and *R* = 80 mm).

With the close proximity of the antenna to the head model, the concern for radiation safety becomes prominent. Hence, the SAR distribution on the head is calculated using the CST software at different frequencies within the frequency range of interest (2.8–4 GHz), with the antenna stimulation power of 0.5 W. The SAR calculations are done with the head model placed at a distance of *D* = 1 and 2 cm from the antenna. The effect of the presence of a tumor inside the head on the SAR distribution of the head is also analyzed. The head model along with the tumor used for the SAR calculation in CST is shown in Figure 9. The tumor is numerically modeled as a sphere of radius 1 cm with a homogeneous region having a dielectric constant of *ɛ*_*r*_ = 50, electric conductivity = 4 S/m, and mass density = 1080 kg/m^3^ [20]. The tumor is placed inside the head at a depth of 1 cm from the head boundary.

**Figure 10:**
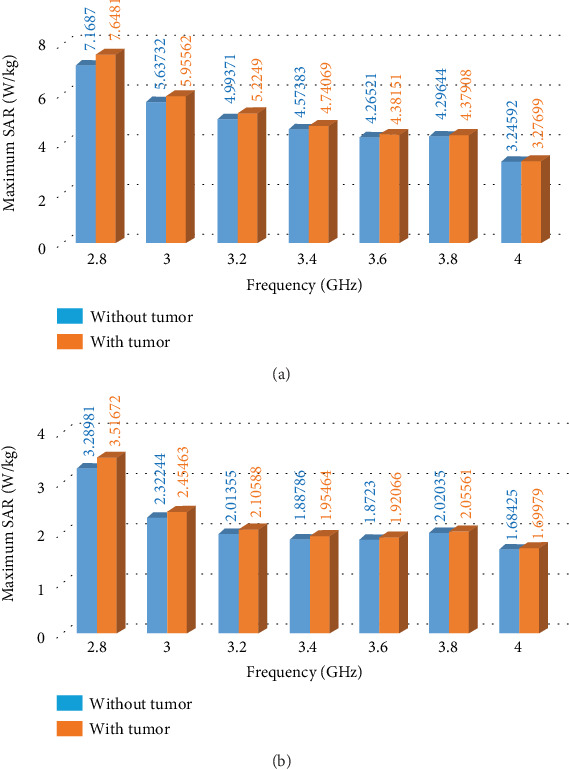
Maximum 10 g averaged SAR on the head model for 0.5 W antenna stimulation power for antenna-to-head distance: (a) *D* = 1 and (b) 2 cm.

The maximum 10 g averaged SAR on the head (with and without the tumor), obtained at different frequencies, is shown in Figure 10. It is observed that the SAR on the head surface increases when a tumor is placed inside the head due to the reflection coming back from the tumor. The increase in SAR is reduced for a larger depth of the tumor into the head; hence, analyses for tumor depth greater than 1 cm is not shown. Again, as the tumor reflections become more obscure as it is placed closer to the head boundary (due to overlapping with the skull reflections), a tumor depth of less than 1 cm is not considered in this work.

The maximum SAR on the head due to the radiation from the antenna should be less than the maximum permissible SAR limit in order to ensure safe exposure to nonionizing radiation. These limits are given in the ICNIRP guidelines [16] for different environments [21]. In Table 1, the dosimetric reference level (DRL) of SAR for local and whole-body exposure is listed for both controlled (restricted) and uncontrolled (unrestricted) environments [21], as specified in the ICNIRP guidelines [16]. It can be seen that the maximum SNR in the head due to exposure in an unrestricted environment should be less than 2 W/kg averaging over 10 g of tissue. IEEE also has defined limits on local exposure reference level (ERL) for controlled/restricted environments as specified in the *IEEE Std C95.1* standards [17].

As per Table 1, it can be said that the maximum SAR on the head due to local exposure in an unrestricted environment should be less than 2 W/kg averaging over 10 g of tissue. Hence, the maximum antenna stimulation power that produces 10 g averaged SAR less than 2 W/kg is calculated at each frequency and listed in Table 2. This calculation is done from the SAR values obtained from Figure 10 (for the case with a tumor) by exploiting the proportional relationship between the SAR and antenna stimulation power.

Here, from Table 2, it can be inferred that the stimulation power to the antenna should not exceed 0.28 W (24.47 dBm) for *D* = 2 cm and 0.13 W (21.14 dBm) for *D* = 1 cm in order to ensure safe radiation exposure to the head model within the entire operating frequency range (2.8–4 GHz).

**Figure 11:**
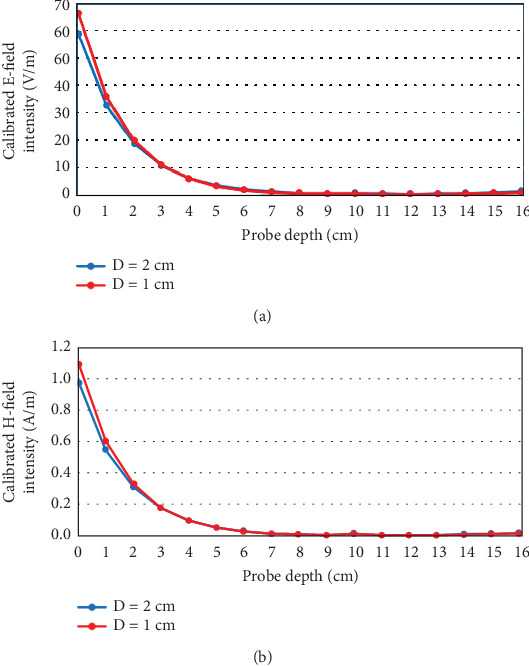
Calibrated E- and H-field intensities at various depths inside the head model for antenna-to-head distance, *D* = 2 and 1 cm, when the antenna stimulation power is kept at the corresponding maximum permissible level.

The E- and H-field intensities presented in Figure 8 are calibrated (i.e., scaled) for the antenna stimulation power of 0.28 W and 0.13 W for *D* = 2 cm and 1 cm, respectively, as per the limits shown in Table 2. The calibrated field intensities are shown in Figure 11, where it can be seen that the field profile remains almost similar after about 3 cm depth inside the head. However, there is a slightly larger field intensity for depths less than 3 cm for *D* = 1 cm as compared to *D* = 2 cm, which can result in larger reflections arising from the skull and shallow regions inside the head.

**Figure 12:**
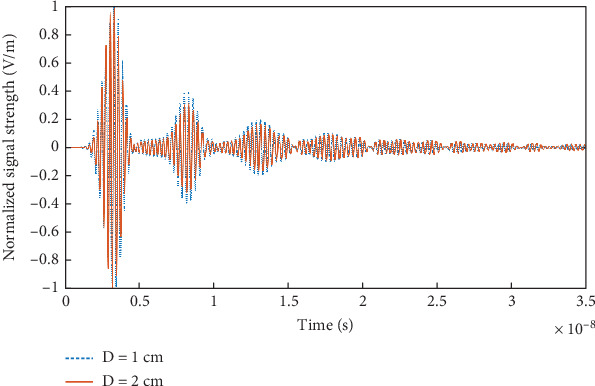
Reflected signal from the head model received by the antenna placed at a distance *D* from the head.

Figure 12 shows the reflected signal in TD received by the antenna when the head model is placed at a distance of *D* = 1 cm and 2 cm. The signal strengths are normalized w.r.t. the corresponding maximum (over time) amplitudes. It is evident that for the case of *D* = 1 cm, larger reverberations are prevalent during the 5–15 ns time interval due to the reflections coming from the head interior. This can cause difficulty in detecting any anomaly close to the skull, within 3 cm depth, as evident from Figure 11, as the reverberations created by the internal reflections from the brain can overlap or obscure the reflections coming from the anomaly (if any). Hence, this fact needs to be emphasized that larger internal reflections/reverberations are received for the case of the antenna-head distance *D* = 1 cm as compared to the case of *D* = 2 cm.

## 4. Antenna Optimization

**Figure 13:**
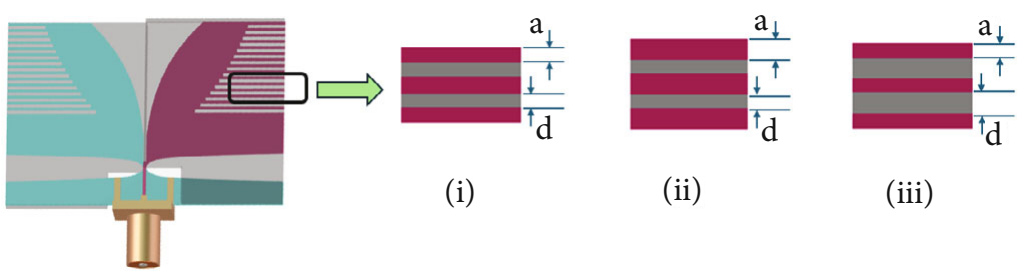
Variation of corrugation profile of the antenna: (i) *a* = 0.5 mm and *d* = 0.5 mm, (ii) *a* = 0.8 mm and *d* = 0.5 mm, and (iii) *a* = 0.5 mm and *d* = 0.8 mm.

Extensive parametric analysis has been presented in [8] to optimize the antenna for the intended application of microwave brain imaging. The effects of different antenna design parameters on the bandwidth and gain of the antenna were investigated for optimization. However, no analysis is carried out to observe the effect of the design parameters on antenna reverberation within the operating frequency band. It has been reported that changing the parameters *L*1, *L*2, *h*1, *h*2, *ws*, *Ls*, *m*, and *p* (as annotated in Figure 1) changes the operating frequency range of the antenna, whereas changing the parameters *a* and *d* (as annotated in Figures 1 and 13) does not significantly alter the operating frequency range of the antenna. Hence, in this work, the effect of the corrugation profile (i.e., parameters *a* and *d*) is analyzed on the reverberation in the received signal within the operating frequency band of the antenna under investigation.

Figure 13 shows the 3 variations in the corrugation profile of the antenna that are analyzed for optimizing the antenna to reduce reverberation for each antenna-to-head distance, D.

**Figure 14:**
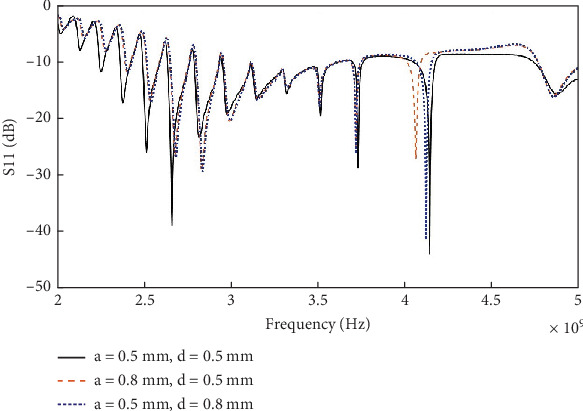
Reflection coefficient (*S*_11_) of the antenna for different variations of corrugation profile.

Figure 14 shows the reflection coefficient plot of the antenna for the three variations of the corrugation profiles. It is evident that changing the values of the parameters *a* and *d* do not significantly alter the bandwidth (*S*_11_ <–10 dB). However, the effect of changing these parameters on the reverberation in the received signal is analyzed in this work.

**Figure 15:**
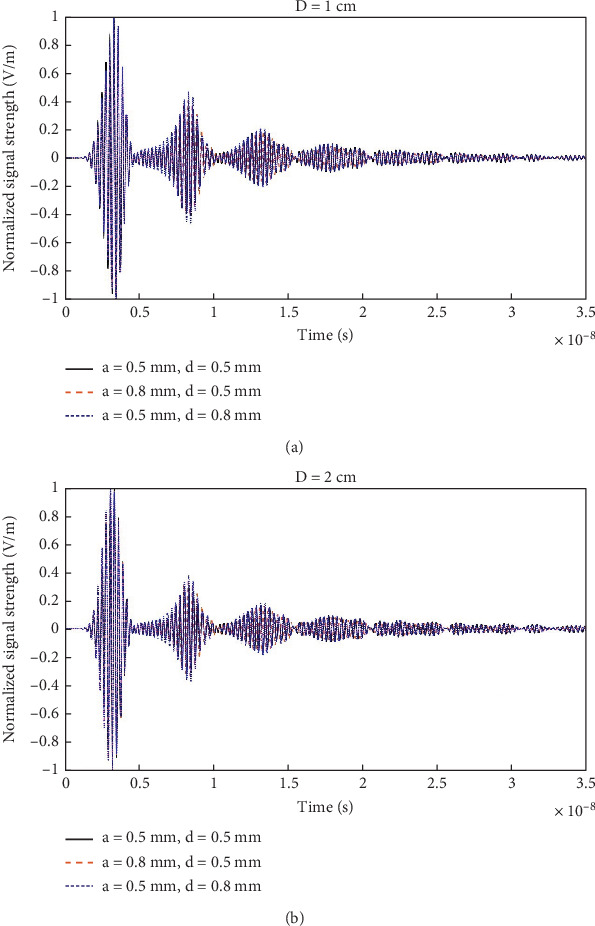
Reflected signal received by the antenna for different variations of corrugation profile: (a) with antenna-to-head distance, *D* = 1 cm, and (b) with antenna-to-head distance, *D* = 2 cm.

**Figure 16:**
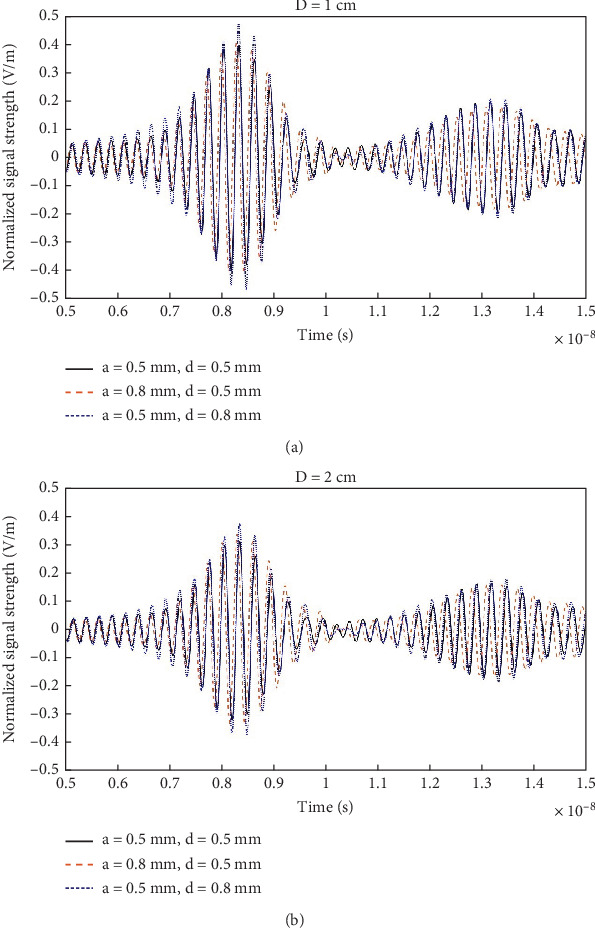
Reflected signal received by the antenna for different variations of corrugation profile with antenna-to-head distance: (a) *D* = 1 cm and (b) *D* = 2 cm.

Figure 15 shows the signal received by the antenna when the head model (without tumor) is placed at a distance of *D* = 1 and 2 cm (as annotated in Figure 5) for different variations of the corrugation profile. The signal strengths are normalized w.r.t. the corresponding maximum (over time) amplitudes. It can be seen that for *a* = 0.5 mm and *d* = 0.8 mm, the reverberation is slightly greater in amplitude than the other variations. This is more clearly observable in the zoomed Figure 16. For the case of *a* = 0.5 mm and *d* = 0.5 mm, the reverberation has the lowest amplitude, especially within 5–15 ns duration, as shown in Figure 16.

## 5. Conclusion

In this paper, an analysis of the effect of the distance of a miniaturized antipodal Vivaldi antenna from a numerical head model for the application of microwave brain imaging is presented. A homogeneous and frequency-nondispersive numerical head model is placed at three different distances from the antenna, and the resulting effects both on the antenna and on the head model are analyzed based on signal receptibility and radiation safety, respectively. The signal receptibility of the antenna is analyzed in terms of antenna surface current distribution and input impedance. The radiation safety of the brain is analyzed by observing the E- and H-field intensities and SAR distribution on the head model. The results are used to calculate the maximum permissible antenna stimulation power to ensure safe local exposure on the head. It can be inferred that as the antenna-head distance increases, the reflected signal incurs more wave spreading loss and gets more contaminated with background noise. On the other hand, as the antenna-head distance is reduced, the skull reflections become more pronounced during the initial portion of the received signal, making it difficult to detect shallow anomalies. Hence, an optimum distance between the antenna and the head should provide a good compromise with respect to the antenna performance and safe exposure considerations. These analyses highlight the point that for a given imaging setup, the optimum antenna-head distance will vary depending on the application-specific antenna, operating frequency range, measurement system, and the surrounding medium. Additionally, analysis of antenna optimization, in terms of antenna reverberation in the received signal, is carried out to ensure noise reduction for each antenna-to-head distance.

## Figures and Tables

**Table 1:** Dosimetric reference level (DRL) from 100 kHz to 6 GHz [16].

**Conditions**	**Persons in unrestricted environments**	**Persons permitted in restricted environments**
	**SAR (W/kg)** ^**a**^	**SAR (W/kg)** ^**a**^
Whole-body exposure	0.08	0.4
Local exposure (head and torso)^b^	2	10

^a^SAR is averaged over 30 min for whole-body exposure and 6 min for local exposure.

^b^Averaged over any 10 g of tissue (defined as a tissue volume in the shape of a cube).

**Table 2:** Maximum permissible antenna stimulation power to obtain less than 2 W/kg 10 g averaged SAR on the head model.

**Frequency (GHz)**	**Maximum permissible antenna stimulation power (W)**	
	**D** = 2 **cm**	**D** = 1 **cm**
2.8	0.28	0.13
3	0.41	0.18
3.2	0.47	0.20
3.4	0.51	0.21
3.6	0.52	0.23
3.8	0.49	0.23
4	0.59	0.31

## Data Availability

The data that support the findings of this study are available from the corresponding author upon reasonable request.
